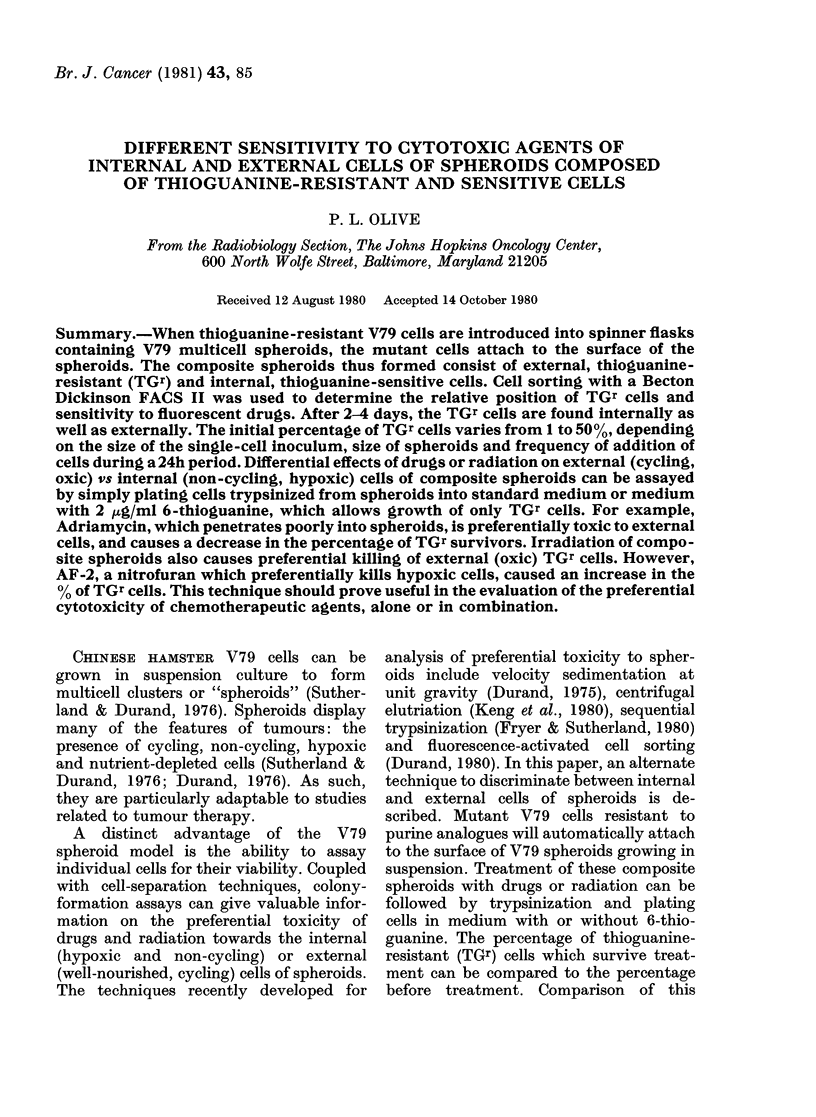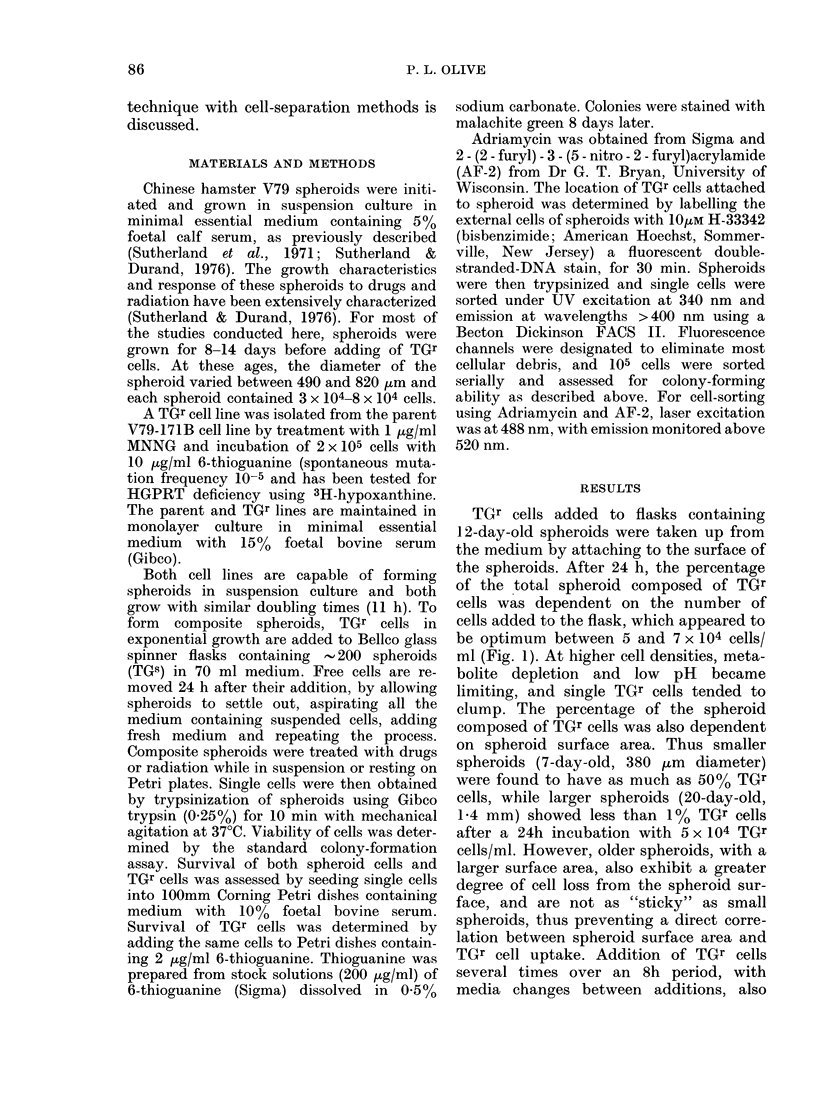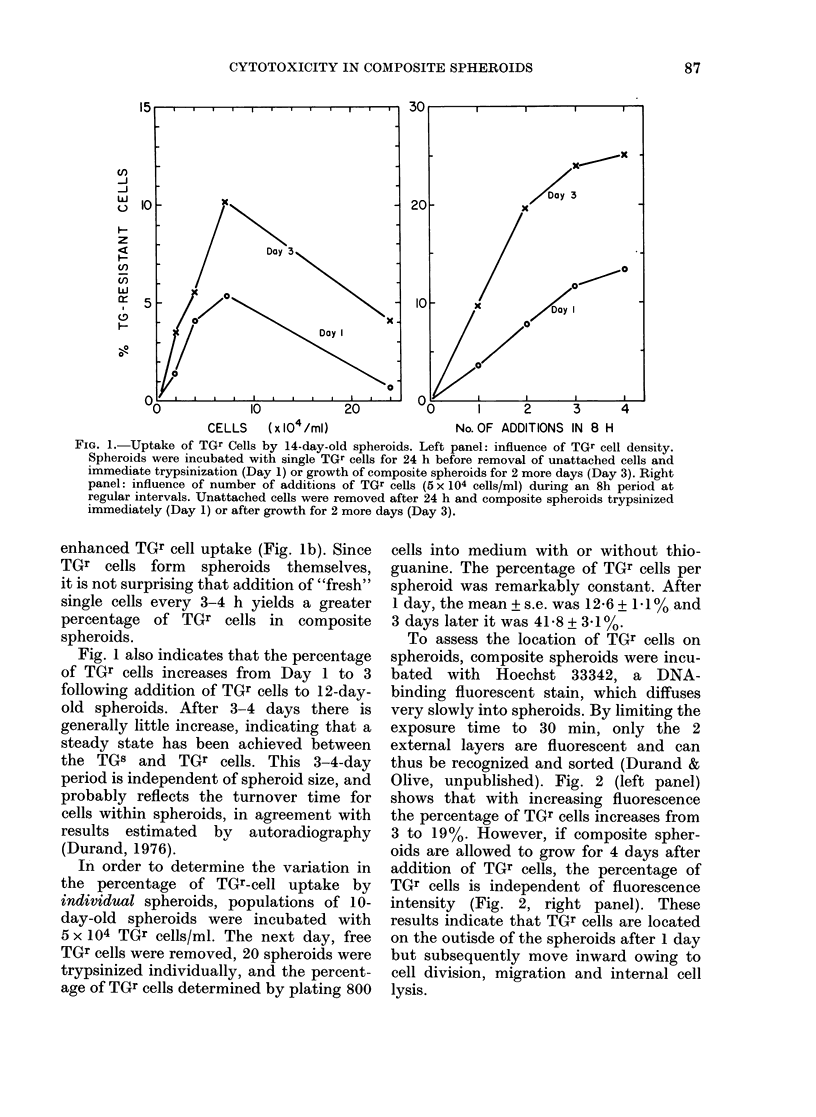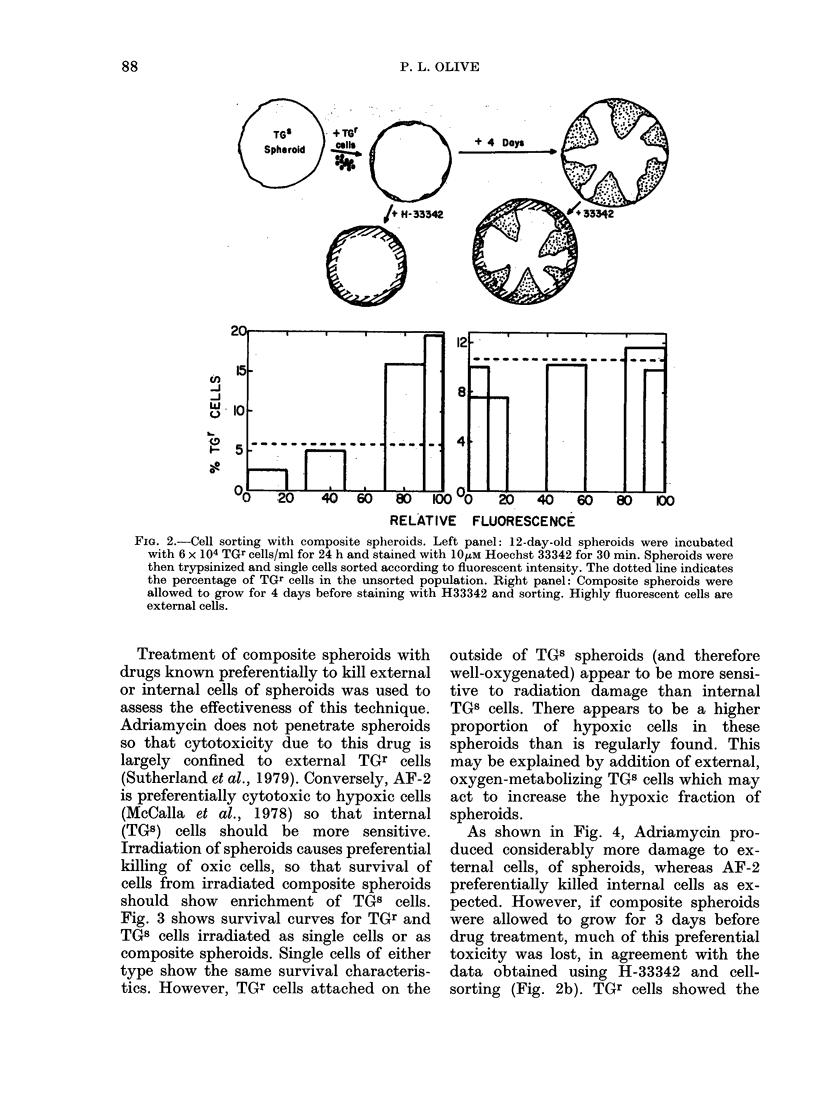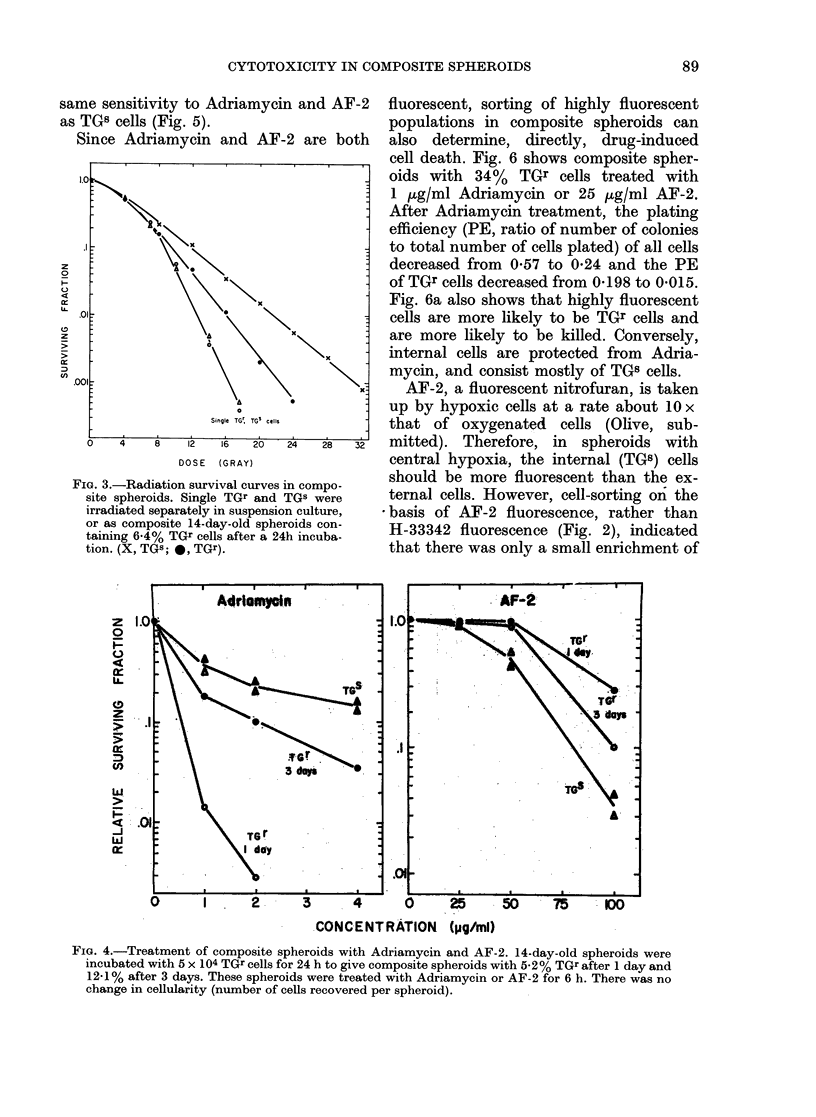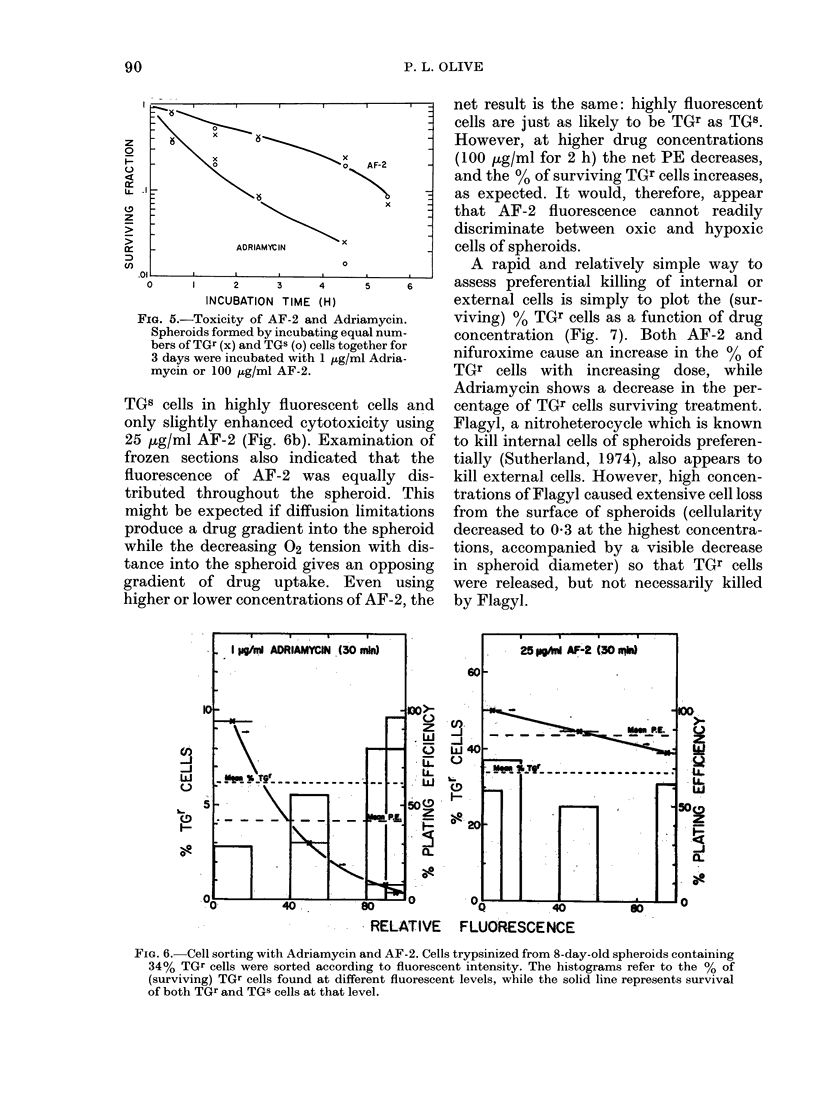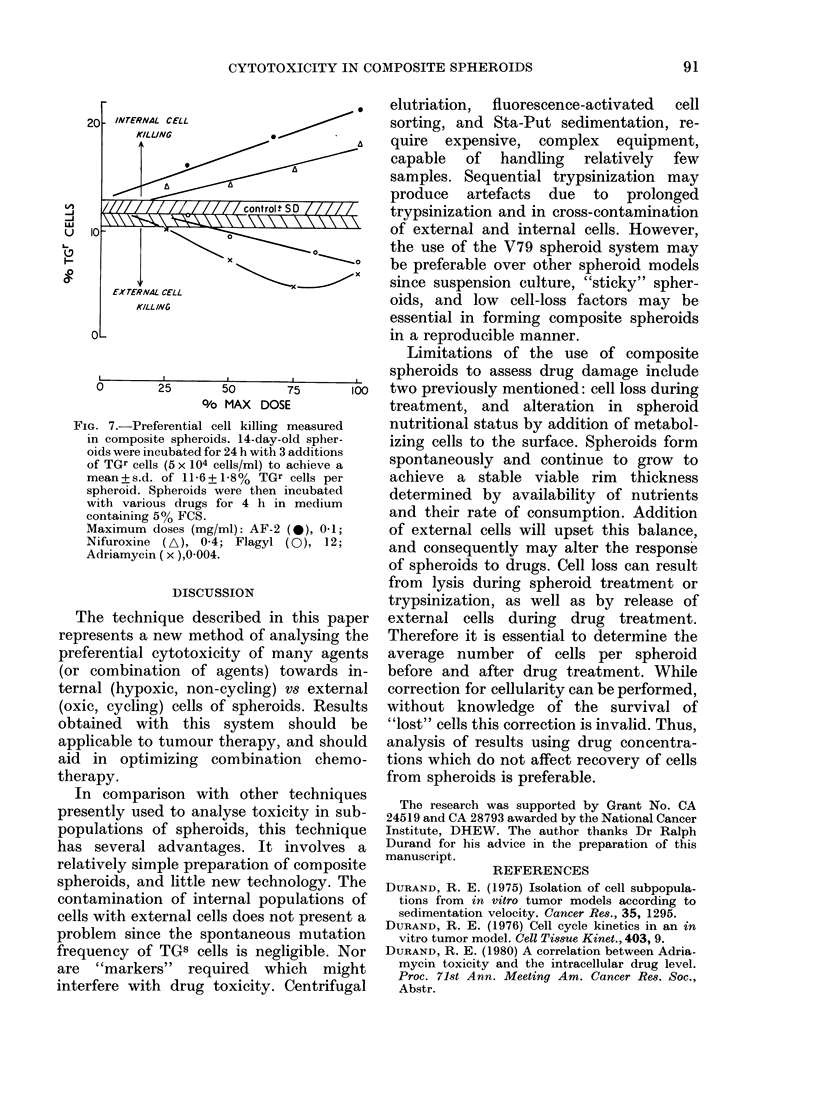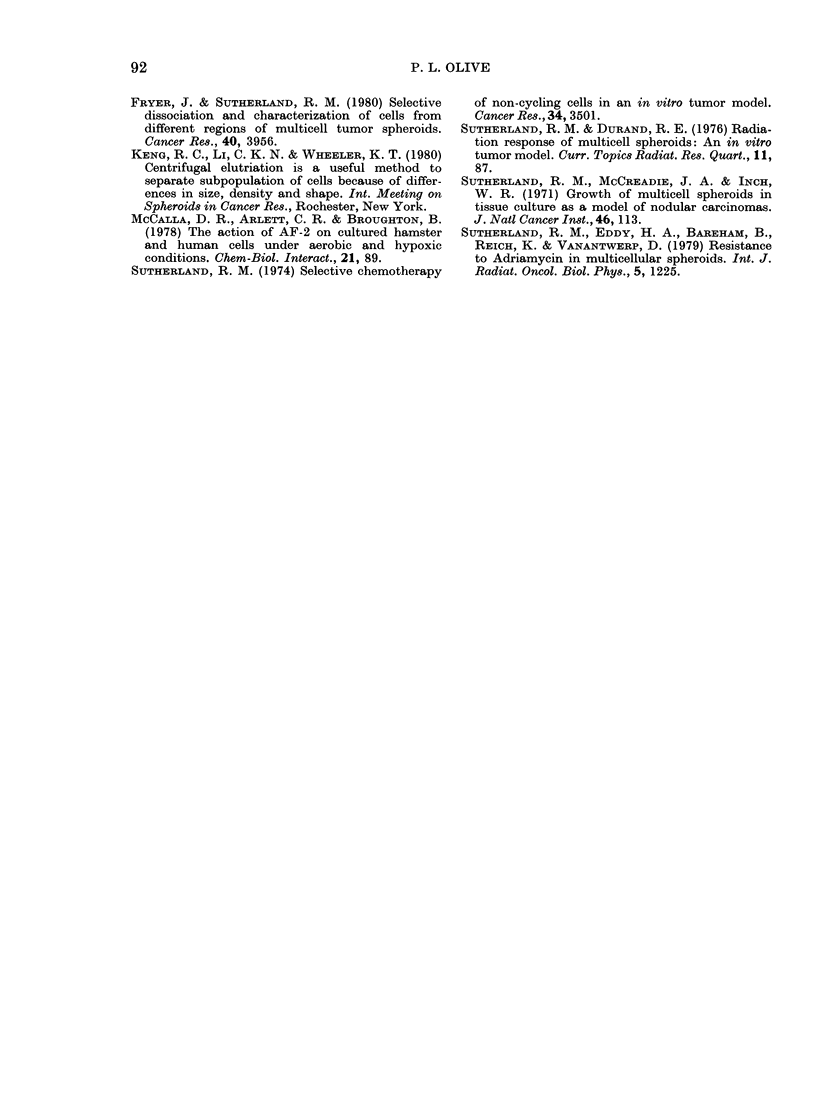# Different sensitivity to cytotoxic agents of internal and external cells of spheroids composed of thioguanine-resistant and sensitive cells.

**DOI:** 10.1038/bjc.1981.12

**Published:** 1981-01

**Authors:** P. L. Olive

## Abstract

When thioguanine-resistant V79 cells are introduced into spinner flasks containing V79 multicell spheroids, the mutant cells attach to the surface of the spheroids. The composite spheroids thus formed consist of external, thioguanine-resistant (TGr) and internal, thioguanine-sensitive cells. Cell sorting with a Becton Dickinson FACS II was used to determine the relative position of TGr cells and sensitivity to fluorescent drugs. After 2-4 days, the TGr cells are found internally as well as externally. The initial percentage of TGr cells varies from 1 to 50%, depending on the size of the single-cell inoculum, size of spheroids and frequency of addition of cells during a 24th period. Differential effects of drugs or radiation on external (cycling, oxic) vs internal (non-cycling, hypoxic) cells of composite spheroids can be assayed by simply plating cells trypsinized from spheroids into standard medium or medium with 2 mu g/ml 6-thioguanine, which allows growth of only TGr cells. For example, Adriamycin, which penetrates poorly into spheroids, is preferentially toxic to external cells, and causes a decrease in the percentage of TGr survivors. Irradiation of composite spheroids also causes preferential killing of external (oxic) TGr cells. However, AF-2, a nitrofuran which preferentially kills hypoxic cells, caused an increase in the 0/0 of TGr cells. This technique should prove useful in the evaluation of the preferential cytotoxicity of chemotherapeutic agents, alone or in combination.


*Br. J. Cancer* (1981) **43**, 85

### DIFFERENT SENSITIVITY TO CYTOTOXIC AGENTS OF INTERNAL AND EXTERNAL CELLS OF SPHEROIDS COMPOSED OF THIOGUANINE-RESISTANT AND SENSITIVE CELLS

P. L. OLIVE

*From the Radiobiology Section, The Johns Hopkins Oncology Center, 600 North Wolfe Street, Baltimore, Maryland 21205*

Received 12 August 1980 Accepted 14 October 1980

**Summary.—When thioguanine-resistant V79 cells are introduced into spinner flasks containing V79 multicell spheroids, the mutant cells attach to the surface of the spheroids. The composite spheroids thus formed consist of external, thioguanine-resistant (TG$^r$) and internal, thioguanine-sensitive cells. Cell sorting with a Becton Dickinson FACS II was used to determine the relative position of TG$^r$ cells and sensitivity to fluorescent drugs. After 2–4 days, the TG$^r$ cells are found internally as well as externally. The initial percentage of TG$^r$ cells varies from 1 to 50%, depending on the size of the single-cell inoculum, size of spheroids and frequency of addition of cells during a 24h period. Differential effects of drugs or radiation on external (cycling, oxic) *vs* internal (non-cycling, hypoxic) cells of composite spheroids can be assayed by simply plating cells trypsinized from spheroids into standard medium or medium with 2 μg/ml 6-thioguanine, which allows growth of only TG$^r$ cells. For example, Adriamycin, which penetrates poorly into spheroids, is preferentially toxic to external cells, and causes a decrease in the percentage of TG$^r$ survivors. Irradiation of composite spheroids also causes preferential killing of external (oxic) TG$^r$ cells. However, AF-2, a nitrofuran which preferentially kills hypoxic cells, caused an increase in the % of TG$^r$ cells. This technique should prove useful in the evaluation of the preferential cytotoxicity of chemotherapeutic agents, alone or in combination.**

Chinese hamster V79 cells can be grown in suspension culture to form multicell clusters or "spheroids" (Sutherland & Durand, 1976). Spheroids display many of the features of tumours: the presence of cycling, non-cycling, hypoxic and nutrient-depleted cells (Sutherland & Durand, 1976; Durand, 1976). As such, they are particularly adaptable to studies related to tumour therapy.

A distinct advantage of the V79 spheroid model is the ability to assay individual cells for their viability. Coupled with cell-separation techniques, colony-formation assays can give valuable information on the preferential toxicity of drugs and radiation towards the internal (hypoxic and non-cycling) or external (well-nourished, cycling) cells of spheroids. The techniques recently developed for analysis of preferential toxicity to spheroids include velocity sedimentation at unit gravity (Durand, 1975), centrifugal elutriation (Keng *et al.*, 1980), sequential trypsinization (Fryer & Sutherland, 1980) and fluorescence-activated cell sorting (Durand, 1980). In this paper, an alternate technique to discriminate between internal and external cells of spheroids is described. Mutant V79 cells resistant to purine analogues will automatically attach to the surface of V79 spheroids growing in suspension. Treatment of these composite spheroids with drugs or radiation can be followed by trypsinization and plating cells in medium with or without 6-thioguanine. The percentage of thioguanine-resistant (TG$^r$) cells which survive treatment can be compared to the percentage before treatment. Comparison of this technique with cell-separation methods is discussed.

### MATERIALS AND METHODS

Chinese hamster V79 spheroids were initiated and grown in suspension culture in minimal essential medium containing 5% foetal calf serum, as previously described (Sutherland *et al.*, 1971; Sutherland & Durand, 1976). The growth characteristics and response of these spheroids to drugs and radiation have been extensively characterized (Sutherland & Durand, 1976). For most of the studies conducted here, spheroids were grown for 8–14 days before adding of $TG^r$ cells. At these ages, the diameter of the spheroid varied between 490 and 820 $\mu$m and each spheroid contained $3 \times 10^4$–$8 \times 10^4$ cells.

A $TG^r$ cell line was isolated from the parent V79-171B cell line by treatment with 1 $\mu$g/ml MNNG and incubation of $2 \times 10^5$ cells with 10 $\mu$g/ml 6-thioguanine (spontaneous mutation frequency $10^{-5}$ and has been tested for HGPRT deficiency using $^3$H-hypoxanthine. The parent and $TG^r$ lines are maintained in monolayer culture in minimal essential medium with 15% foetal bovine serum (Gibco).

Both cell lines are capable of forming spheroids in suspension culture and both grow with similar doubling times (11 h). To form composite spheroids, $TG^r$ cells in exponential growth are added to Bellco glass spinner flasks containing ~200 spheroids ($TG^s$) in 70 ml medium. Free cells are removed 24 h after their addition, by allowing spheroids to settle out, aspirating all the medium containing suspended cells, adding fresh medium and repeating the process. Composite spheroids were treated with drugs or radiation while in suspension or resting on Petri plates. Single cells were then obtained by trypsinization of spheroids using Gibco trypsin (0·25%) for 10 min with mechanical agitation at 37°C. Viability of cells was determined by the standard colony-formation assay. Survival of both spheroid cells and $TG^r$ cells was assessed by seeding single cells into 100mm Corning Petri dishes containing medium with 10% foetal bovine serum. Survival of $TG^r$ cells was determined by adding the same cells to Petri dishes containing 2 $\mu$g/ml 6-thioguanine. Thioguanine was prepared from stock solutions (200 $\mu$g/ml) of 6-thioguanine (Sigma) dissolved in 0·5% sodium carbonate. Colonies were stained with malachite green 8 days later.

Adriamycin was obtained from Sigma and 2-(2-furyl)-3-(5-nitro-2-furyl)acrylamide (AF-2) from Dr G. T. Bryan, University of Wisconsin. The location of $TG^r$ cells attached to spheroid was determined by labelling the external cells of spheroids with 10$\mu$M H-33342 (bisbenzimide; American Hoechst, Sommerville, New Jersey) a fluorescent double-stranded-DNA stain, for 30 min. Spheroids were then trypsinized and single cells were sorted under UV excitation at 340 nm and emission at wavelengths >400 nm using a Becton Dickinson FACS II. Fluorescence channels were designated to eliminate most cellular debris, and $10^5$ cells were sorted serially and assessed for colony-forming ability as described above. For cell-sorting using Adriamycin and AF-2, laser excitation was at 488 nm, with emission monitored above 520 nm.

### RESULTS

$TG^r$ cells added to flasks containing 12-day-old spheroids were taken up from the medium by attaching to the surface of the spheroids. After 24 h, the percentage of the total spheroid composed of $TG^r$ cells was dependent on the number of cells added to the flask, which appeared to be optimum between 5 and $7 \times 10^4$ cells/ml (Fig. 1). At higher cell densities, metabolite depletion and low pH became limiting, and single $TG^r$ cells tended to clump. The percentage of the spheroid composed of $TG^r$ cells was also dependent on spheroid surface area. Thus smaller spheroids (7-day-old, 380 $\mu$m diameter) were found to have as much as 50% $TG^r$ cells, while larger spheroids (20-day-old, 1·4 mm) showed less than 1% $TG^r$ cells after a 24h incubation with $5 \times 10^4$ $TG^r$ cells/ml. However, older spheroids, with a larger surface area, also exhibit a greater degree of cell loss from the spheroid surface, and are not as "sticky" as small spheroids, thus preventing a direct correlation between spheroid surface area and $TG^r$ cell uptake. Addition of $TG^r$ cells several times over an 8h period, with media changes between additions, also

FIG. 1.—Uptake of TG$^r$ Cells by 14-day-old spheroids. Left panel: influence of TG$^r$ cell density. Spheroids were incubated with single TG$^r$ cells for 24 h before removal of unattached cells and immediate trypsinization (Day 1) or growth of composite spheroids for 2 more days (Day 3). Right panel: influence of number of additions of TG$^r$ cells ($5 \times 10^4$ cells/ml) during an 8h period at regular intervals. Unattached cells were removed after 24 h and composite spheroids trypsinized immediately (Day 1) or after growth for 2 more days (Day 3).

enhanced TG$^r$ cell uptake (Fig. 1b). Since TG$^r$ cells form spheroids themselves, it is not surprising that addition of "fresh" single cells every 3–4 h yields a greater percentage of TG$^r$ cells in composite spheroids.

Fig. 1 also indicates that the percentage of TG$^r$ cells increases from Day 1 to 3 following addition of TG$^r$ cells to 12-day-old spheroids. After 3–4 days there is generally little increase, indicating that a steady state has been achieved between the TG$^s$ and TG$^r$ cells. This 3–4-day period is independent of spheroid size, and probably reflects the turnover time for cells within spheroids, in agreement with results estimated by autoradiography (Durand, 1976).

In order to determine the variation in the percentage of TG$^r$-cell uptake by *individual* spheroids, populations of 10-day-old spheroids were incubated with $5 \times 10^4$ TG$^r$ cells/ml. The next day, free TG$^r$ cells were removed, 20 spheroids were trypsinized individually, and the percentage of TG$^r$ cells determined by plating 800 cells into medium with or without thioguanine. The percentage of TG$^r$ cells per spheroid was remarkably constant. After 1 day, the mean ± s.e. was 12·6 ± 1·1% and 3 days later it was 41·8 ± 3·1%.

To assess the location of TG$^r$ cells on spheroids, composite spheroids were incubated with Hoechst 33342, a DNA-binding fluorescent stain, which diffuses very slowly into spheroids. By limiting the exposure time to 30 min, only the 2 external layers are fluorescent and can thus be recognized and sorted (Durand & Olive, unpublished). Fig. 2 (left panel) shows that with increasing fluorescence the percentage of TG$^r$ cells increases from 3 to 19%. However, if composite spheroids are allowed to grow for 4 days after addition of TG$^r$ cells, the percentage of TG$^r$ cells is independent of fluorescence intensity (Fig. 2, right panel). These results indicate that TG$^r$ cells are located on the outisde of the spheroids after 1 day but subsequently move inward owing to cell division, migration and internal cell lysis.

FIG. 2.—Cell sorting with composite spheroids. Left panel: 12-day-old spheroids were incubated with $6 \times 10^4$ TG$^r$ cells/ml for 24 h and stained with 10$\mu$M Hoechst 33342 for 30 min. Spheroids were then trypsinized and single cells sorted according to fluorescent intensity. The dotted line indicates the percentage of TG$^r$ cells in the unsorted population. Right panel: Composite spheroids were allowed to grow for 4 days before staining with H33342 and sorting. Highly fluorescent cells are external cells.

Treatment of composite spheroids with drugs known preferentially to kill external or internal cells of spheroids was used to assess the effectiveness of this technique. Adriamycin does not penetrate spheroids so that cytotoxicity due to this drug is largely confined to external TG$^r$ cells (Sutherland *et al.*, 1979). Conversely, AF-2 is preferentially cytotoxic to hypoxic cells (McCalla *et al.*, 1978) so that internal (TG$^s$) cells should be more sensitive. Irradiation of spheroids causes preferential killing of oxic cells, so that survival of cells from irradiated composite spheroids should show enrichment of TG$^s$ cells. Fig. 3 shows survival curves for TG$^r$ and TG$^s$ cells irradiated as single cells or as composite spheroids. Single cells of either type show the same survival characteristics. However, TG$^r$ cells attached on the outside of TG$^s$ spheroids (and therefore well-oxygenated) appear to be more sensitive to radiation damage than internal TG$^s$ cells. There appears to be a higher proportion of hypoxic cells in these spheroids than is regularly found. This may be explained by addition of external, oxygen-metabolizing TG$^s$ cells which may act to increase the hypoxic fraction of spheroids.

As shown in Fig. 4, Adriamycin produced considerably more damage to external cells, of spheroids, whereas AF-2 preferentially killed internal cells as expected. However, if composite spheroids were allowed to grow for 3 days before drug treatment, much of this preferential toxicity was lost, in agreement with the data obtained using H-33342 and cell-sorting (Fig. 2b). TG$^r$ cells showed the same sensitivity to Adriamycin and AF-2 as TG$^s$ cells (Fig. 5).

Since Adriamycin and AF-2 are both

FIG. 3.—Radiation survival curves in composite spheroids. Single TG$^r$ and TG$^s$ were irradiated separately in suspension culture, or as composite 14-day-old spheroids containing 6·4% TG$^r$ cells after a 24h incubation. (X, TG$^s$; ●, TG$^r$).

fluorescent, sorting of highly fluorescent populations in composite spheroids can also determine, directly, drug-induced cell death. Fig. 6 shows composite spheroids with 34% TG$^r$ cells treated with 1 $\mu$g/ml Adriamycin or 25 $\mu$g/ml AF-2. After Adriamycin treatment, the plating efficiency (PE, ratio of number of colonies to total number of cells plated) of all cells decreased from 0·57 to 0·24 and the PE of TG$^r$ cells decreased from 0·198 to 0·015. Fig. 6a also shows that highly fluorescent cells are more likely to be TG$^r$ cells and are more likely to be killed. Conversely, internal cells are protected from Adriamycin, and consist mostly of TG$^s$ cells.

AF-2, a fluorescent nitrofuran, is taken up by hypoxic cells at a rate about 10 × that of oxygenated cells (Olive, submitted). Therefore, in spheroids with central hypoxia, the internal (TG$^s$) cells should be more fluorescent than the external cells. However, cell-sorting on the basis of AF-2 fluorescence, rather than H-33342 fluorescence (Fig. 2), indicated that there was only a small enrichment of

FIG. 4.—Treatment of composite spheroids with Adriamycin and AF-2. 14-day-old spheroids were incubated with 5 x 10$^4$ TG$^r$ cells for 24 h to give composite spheroids with 5·2% TG$^r$ after 1 day and 12·1% after 3 days. These spheroids were treated with Adriamycin or AF-2 for 6 h. There was no change in cellularity (number of cells recovered per spheroid).

FIG. 5.—Toxicity of AF-2 and Adriamycin. Spheroids formed by incubating equal numbers of $TG^r$ (x) and $TG^s$ (o) cells together for 3 days were incubated with 1 μg/ml Adriamycin or 100 μg/ml AF-2.

$TG^s$ cells in highly fluorescent cells and only slightly enhanced cytotoxicity using 25 μg/ml AF-2 (Fig. 6b). Examination of frozen sections also indicated that the fluorescence of AF-2 was equally distributed throughout the spheroid. This might be expected if diffusion limitations produce a drug gradient into the spheroid while the decreasing $O_2$ tension with distance into the spheroid gives an opposing gradient of drug uptake. Even using higher or lower concentrations of AF-2, the net result is the same: highly fluorescent cells are just as likely to be $TG^r$ as $TG^s$. However, at higher drug concentrations (100 μg/ml for 2 h) the net PE decreases, and the % of surviving $TG^r$ cells increases, as expected. It would, therefore, appear that AF-2 fluorescence cannot readily discriminate between oxic and hypoxic cells of spheroids.

A rapid and relatively simple way to assess preferential killing of internal or external cells is simply to plot the (surviving) % $TG^r$ cells as a function of drug concentration (Fig. 7). Both AF-2 and nifuroxime cause an increase in the % of $TG^r$ cells with increasing dose, while Adriamycin shows a decrease in the percentage of $TG^r$ cells surviving treatment. Flagyl, a nitroheterocycle which is known to kill internal cells of spheroids preferentially (Sutherland, 1974), also appears to kill external cells. However, high concentrations of Flagyl caused extensive cell loss from the surface of spheroids (cellularity decreased to 0·3 at the highest concentrations, accompanied by a visible decrease in spheroid diameter) so that $TG^r$ cells were released, but not necessarily killed by Flagyl.

FIG. 6.—Cell sorting with Adriamycin and AF-2. Cells trypsinized from 8-day-old spheroids containing 34% $TG^r$ cells were sorted according to fluorescent intensity. The histograms refer to the % of (surviving) $TG^r$ cells found at different fluorescent levels, while the solid line represents survival of both $TG^r$ and $TG^s$ cells at that level.

FIG. 7.—Preferential cell killing measured in composite spheroids. 14-day-old spheroids were incubated for 24 h with 3 additions of TG$^r$ cells ($5 \times 10^4$ cells/ml) to achieve a mean ± s.d. of 11·6 ± 1·8% TG$^r$ cells per spheroid. Spheroids were then incubated with various drugs for 4 h in medium containing 5% FCS.
Maximum doses (mg/ml): AF-2 (●), 0·1; Nifuroxine (△), 0·4; Flagyl (○), 12; Adriamycin ( × ),0·004.

### DISCUSSION

The technique described in this paper represents a new method of analysing the preferential cytotoxicity of many agents (or combination of agents) towards internal (hypoxic, non-cycling) *vs* external (oxic, cycling) cells of spheroids. Results obtained with this system should be applicable to tumour therapy, and should aid in optimizing combination chemotherapy.

In comparison with other techniques presently used to analyse toxicity in subpopulations of spheroids, this technique has several advantages. It involves a relatively simple preparation of composite spheroids, and little new technology. The contamination of internal populations of cells with external cells does not present a problem since the spontaneous mutation frequency of TG$^s$ cells is negligible. Nor are "markers" required which might interfere with drug toxicity. Centrifugal elutriation, fluorescence-activated cell sorting, and Sta-Put sedimentation, require expensive, complex equipment, capable of handling relatively few samples. Sequential trypsinization may produce artefacts due to prolonged trypsinization and in cross-contamination of external and internal cells. However, the use of the V79 spheroid system may be preferable over other spheroid models since suspension culture, "sticky" spheroids, and low cell-loss factors may be essential in forming composite spheroids in a reproducible manner.

Limitations of the use of composite spheroids to assess drug damage include two previously mentioned: cell loss during treatment, and alteration in spheroid nutritional status by addition of metabolizing cells to the surface. Spheroids form spontaneously and continue to grow to achieve a stable viable rim thickness determined by availability of nutrients and their rate of consumption. Addition of external cells will upset this balance, and consequently may alter the response of spheroids to drugs. Cell loss can result from lysis during spheroid treatment or trypsinization, as well as by release of external cells during drug treatment. Therefore it is essential to determine the average number of cells per spheroid before and after drug treatment. While correction for cellularity can be performed, without knowledge of the survival of "lost" cells this correction is invalid. Thus, analysis of results using drug concentrations which do not affect recovery of cells from spheroids is preferable.

The research was supported by Grant No. CA 24519 and CA 28793 awarded by the National Cancer Institute, DHEW. The author thanks Dr Ralph Durand for his advice in the preparation of this manuscript.

### REFERENCES

DURAND, R. E. (1975) Isolation of cell subpopulations from *in vitro* tumor models according to sedimentation velocity. *Cancer Res.*, **35,** 1295.

DURAND, R. E. (1976) Cell cycle kinetics in an *in* vitro tumor model. *Cell Tissue Kinet.*, **403,** 9.

DURAND, R. E. (1980) A correlation between Adriamycin toxicity and the intracellular drug level. *Proc. 71st Ann. Meeting Am. Cancer Res. Soc.*, Abstr.

FRYER, J. & SUTHERLAND, R. M. (1980) Selective dissociation and characterization of cells from different regions of multicell tumor spheroids. *Cancer Res.*, **40**, 3956.

KENG, R. C., LI, C. K. N. & WHEELER, K. T. (1980) Centrifugal elutriation is a useful method to separate subpopulation of cells because of differences in size, density and shape. *Int. Meeting on Spheroids in Cancer Res.*, Rochester, New York.

McCALLA, D. R., ARLETT, C. R. & BROUGHTON, B. (1978) The action of AF-2 on cultured hamster and human cells under aerobic and hypoxic conditions. *Chem-Biol. Interact.*, **21**, 89.

SUTHERLAND, R. M. (1974) Selective chemotherapy of non-cycling cells in an *in vitro* tumor model. *Cancer Res.*, **34**, 3501.

SUTHERLAND, R. M. & DURAND, R. E. (1976) Radiation response of multicell spheroids: An *in vitro* tumor model. *Curr. Topics Radiat. Res. Quart.*, **11**, 87.

SUTHERLAND, R. M., McCREADIE, J. A. & INCH, W. R. (1971) Growth of multicell spheroids in tissue culture as a model of nodular carcinomas. *J. Natl Cancer Inst.*, **46**, 113.

SUTHERLAND, R. M., EDDY, H. A., BAREHAM, B., REICH, K. & VANANTWERP, D. (1979) Resistance to Adriamycin in multicellular spheroids. *Int. J. Radiat. Oncol. Biol. Phys.*, **5**, 1225.